# Role of Heat Expansion with a Series of Ionic Liquids: The Case for Isochoric Thermoelectric Generators and Minimal Steric Repulsion

**DOI:** 10.3390/e21111086

**Published:** 2019-11-06

**Authors:** Marcus Jackson, Robert R Engel, Luat T. Vuong

**Affiliations:** 1 Department of Chemistry, Queens College of the City University of New York, Flushing, NY 11367, USA; marcus1997jackson@gmail.com (M.J.); robert.engel@quatcare.org (R.R.E.); 2 Department of Physics, Queens College of the City University of New York, Flushing, NY 11367, USA; 3 Department of Mechanical Engineering, University of California at Riverside, Riverside, CA 92521, USA

**Keywords:** thermoelectric, Seebeck coefficient, convective thermoelectric generator, complex ionic-liquid systems, steric forces

## Abstract

The role of convection in liquid thermoelectric cells may be difficult to predict because the inter- and intra-molecular interactions are not currently incorporated into thermodynamic models. Here, we study the thermoelectric response of a series of five anhydrous 1-methyl-3-alkylimidazolium halide ionic liquids with varied chain length and counterion in a high-aspect-ratio, horizontal-temperature-gradient geometry, where convection is minimal. While a canonical, constant-volume thermodynamic model indicates that greater disparity and longer aliphatic groups should exhibit larger Seebeck coefficients, we instead measure the opposite: Longer aliphatic chains correlate with lower densities and greater heat expansion, stronger intermolecular associations, stronger steric repulsion, and lower Seebeck coefficients. As evidence of the critical role of thermal expansion, we measure that the Seebeck effect is nonlinear: Values of −2.8 mV/K with a 10 K temperature difference and −1.8 mV/K with a 50 K difference are measured with ether ion. Our results indicate that steric repulsion and heat expansion are important considerations in ionic liquid design; with large temperature differences, the Seebeck coefficient correlates negatively with heat expansion. Our results suggest that Seebeck values may improve if thermal expansion and steric forces are limited in a pressurized, isochoric, convection-free design.

## 1. Introduction

There is currently significant interest in the development of thermoelectric generators (TEGs) that employ multi-phase fluids. The thermoelectric charging of electrodes [1] is governed largely by thermophoresis, i.e., thermodiffusion, thermomigration, or a Soret effect of complementary-charged species [2], which leads to the presence of an electrical potential across a temperature gradient. It is often reported that liquid TEGs show promise for harvesting waste heat or thermal energy without the limitations or material engineering associated with the Wiedemann–Franz law [3]; however, the ideal geometric designs of TEGs are still challenging to model and optimize a priori, which is related to the complex TEG fluid behavior. The TEG multi-phase fluids are generally composed of complementary asymmetric charged species and their physical properties vary widely. Room temperature ionic liquids (RTILs) [4] are composed solely of complementary ions. In contrast, electrolytes and nanofluids contain charged nanoparticles and ions that are dispersed in solvent [5,6].

Recently, there has been a trend towards multi-phase liquid TEG designs that exhibit higher convection. The presence of convection appears to amplify the thermoelectric response and increase the rate of electrode recharging [7,8,9]. Magnetic nanofluids have been shown to exhibit enhanced potentials with increased convection in the presence of externally applied magnetic fields [10]. However, with silver nanofluids, the presence of radiation and evaporation indicate that the role of convection should be scrutinized [11]. In addition, with ionic liquids undergoing laminar flow, the corresponding changes in physicochemical properties of a moving ionic liquid may reduce charge extraction at an electrode [12]. Thermodiffusion and thermophoresis, alone, is a process whose sign or direction can change depending on the local chemical environment [13]. The physical parameters central to the TEG fluid models such as viscosity, charge mobility, and charge collection efficiency depend critically on the intermolecular interactions of the fluid [14,15,16].

Here, we show that intra-molecular behaviors carry an outsized role in the macroscopic fluid dynamics of TEGs as we study the thermoelectric charging of a range of viscous, RTILs. As pure molten salts, RTILs contain cations and anions of different sizes and charge distributions, so that strong, frustrated Coulombic interactions translate to low volatility and high boiling points. RTILs also exhibit high and temperature-dependent viscosities that are 2-4 orders of magnitude greater than water [17]. Since most ionic liquids remain in the liquid phase over hundreds of Kelvin [12,18,19] and since there has been significant progress towards the low-cost production of imidazolium-based ionic liquids [20], RTILs may be a viable and robust liquid in TEG applications over an extreme range of temperatures.

With a minimal-convection geometry, we demonstrate that the Seebeck coefficient correlates negatively with heat expansion for RTILs. Since the temperature-dependent changes in the viscosity and convection are amplified with larger heat expansion coefficients, knowledge of the isolated role that heat expansion plays is essential for understanding TEG design. Theoretically, with a canonical, constant-volume analysis, it is the longer aliphatic groups that should exhibit larger Seebeck coefficients; however, we measure the opposite. With longer aliphatic chains, the inter-molecular associations are reduced due to steric repulsion, which correlates with lower densities and greater heat expansion. We observe Seebeck values of up to −2.8 mV/K with a corresponding 6 K difference between the terminals and −1.8 mV/K at a 50 K difference corresponding with the ether-ion samples. Our results indicate that by minimizing or limiting the thermal expansion of ionic liquids, the efficiency of liquid-state convection-free TEGs may be improved. In addition, the effect of intra-molecular forces in ionic liquids warrant further attention in the design of TEG fluids.

## 2. Materials and Methods

**Figure 1:**
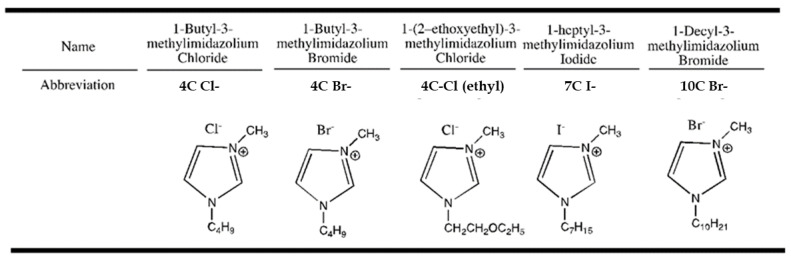
Chemical nomenclature and structures of room temperature ionic liquids (RTILs) used in this study.

Five anhydrous 1-methyl-3-alkylimidazolium halides are synthesized with varying chain length and counterion and are shown in Figure 1. The preparation of the ionic liquids involved in this study are performed by a well established method (anion exchange). Purification of the ionic liquid materials is completed by dissolution of the isolated material in an anhydrous organic solvent (acetonitrile), and passage of the solution through a silica gel column [21]. In this way, the last traces of water are removed. Purity is established both by 1H NMR spectrometry, which provided a verification of the structure, and by elemental analyses (C,H,N), which established the purity of the material and the absence of materials of other formulations. All materials isolated in this way exhibited 1H and 13C NMR spectra in accord with the indicated structures. Other approaches toward preparation of ionic liquids that have been established in more recent times are might be useful for further exploration [22,23,24]. There exists wide capability to fine-tune the physical properties by simply changing the structure of the cation or the counterion [25].

**Figure 2:**
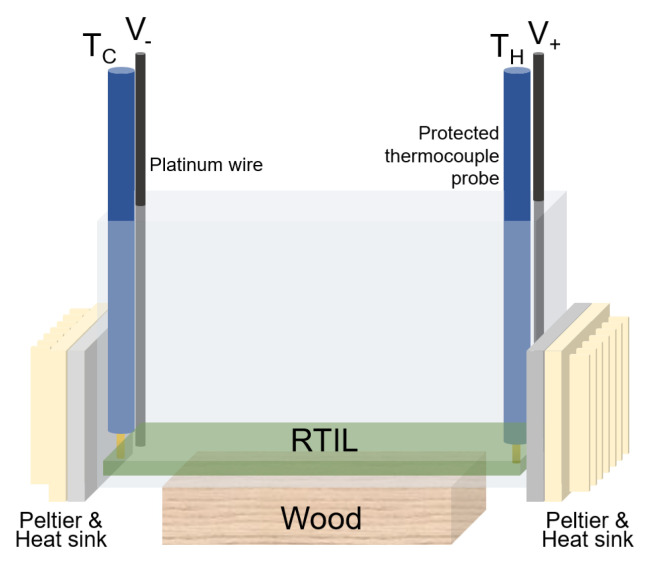
Experimental setup. The room-temperature ionic liquid (RTIL) fills only a few millimeters of the bottom of the quartz cuvette. The cold and hot temperatures $T_{C}$ and $T_{H}$ determine the polarity of the measured voltage. Peltier coolers and heat sinks are afixed with thermal paste.

The schematic in Figure 2 illustrates the TE set-up used for Seebeck coefficient measurements. Convection is expected to be minimal since the temperature gradient is directed in the horizontal direction and because pressure changes in vertical direction associated with gravity are small since the liquid sample is shallow. Each RTIL sample is tested in a glass cell (10 cm × 0.5 cm × 4 cm) situated between two TECI-12706 Peltier devices (4 cm × 4 cm × 0.34 cm) with heat sinks and thermal paste to prevent overheating. Approximately 2 mL of RTIL is placed in the container, which covers the cuvette bottom with a 0.4 cm layer. To prevent conductive heat from leaving the system through the bottom of the cuvette, a wooden block is placed below the cell.

The voltage Δϕ is measured in this high-aspect ratio, minimal convection, negligible current geometry. Platinum wire electrodes (3-cm length, 0.5-mm diameter) are used to measure the voltage difference across the RTIL. An input voltage is supplied (TenMATA Laboratory DC Power Supply 72-2080) to the Peltier devices to generate a temperature difference across the RTIL. All measurements displayed by the voltmeter are relative values between electrodes [26]. One terminal is held constant at 23 K as the temperature at the other electrode varies 293–343 K. Our RTILs are viscous and exhibit low convection as measured by negligible and fluctuating electrical currents on the order of microamperes. To prevent redox contamination, regions of the electrode that are not submerged in fluid are wrapped in parafilm. The temperature difference across the RTIL is measured with two thermocouples (Omega HH309a Datalogger Thermometer). Voltage and temperature measurements are recorded simultaneously.

To measure the heat expansion coefficient, the ionic liquid is heated on a hot plate in a graduated cylinder. When heated, the RTIL expands. The volume is measured visually. The heat expansion coefficient is equal to the change in volume divided by the average volume between the two temperatures.

## 3. Results and Analysis

**Figure 3:**
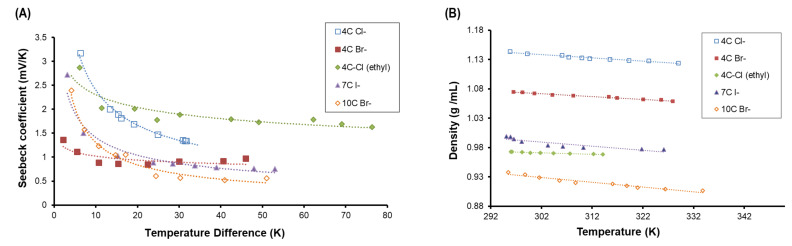
(**A**) Measured voltages as a function of temperature difference between electrodes for a series of ionic liquids. (**B**) RTIL density vs. temperature and linear fit.

The measured voltages as a function of temperature differences are shown in Figure 3A. Seebeck coefficients larger than −3 mV/K are measured for small temperature differences and drops significantly when measured for larger temperature differences >10 K and stabilizes with larger temperature differences. The temperature-dependent Seebeck effect in our experiment is similar to the nonlinear Soret coefficient reported by [27], which represents gradual miscibility above a lower consolute or critical point.

With this data alone, it is difficult to discern the definitive role of RTIL chain length. Longer aliphatic chains correlate with the production of smaller voltages; however, the drop in Seebeck values at lower temperatures is sharper. The role of counter ion and ether ion for the 3-length RTIL is also not clear. Therefore, the challenge in our analysis lies in identifying a trend to understand these results. We infer that thermal expansion carries a role. The density of the RTILs is measured as a function of temperature and shown to follow a linear fit (Figure 3B). Density exhibits an inverse relationship with chain length, which would be explained by the steric effect that inhibits proper packing of mass per unit volume via electron repulsion.

The common analytic relation for the thermoelectric behavior is derived from the free energy gradients produced by entropy per unit charge across a temperature gradient [5,28]

(1) J=−σ(V−αT),

where *J* is the current density, σ is the electrical conductivity, *V* is the voltage or potential difference between electrodes, α is the Seebeck coefficient, and *T* is the temperature difference between electrodes.

When steady-state conditions are assumed or when J≈0, then the Seebeck coefficient is expressed:

(2) $$\alpha =-\frac{\Delta \phi }{\Delta T}.$$

We emphasize that this linear relationship between temperature and voltage is an approximation. Since the measured voltages in our experiments depend on the temperature difference and the Seebeck coefficient α is difficult to fit to Equation (2), in order to calculate a relation for α, we infer a linear fit between the measured voltage and temperature difference by considering a strong voltage offset $\phi_{0}$, or

(3) $$\alpha_{lin}=-\frac{\Delta \phi -\phi_{0}}{\Delta T}.$$

**Figure 4:**
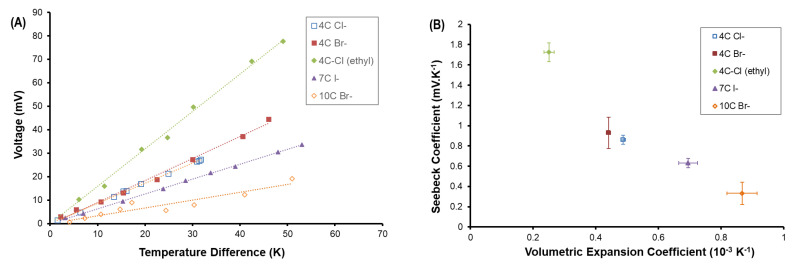
(**A**) Linearized changes in voltages as a function of temperature difference in order to estimate a linear Seebeck coefficient; (**B**) Negative correlation between volumetric heat expansion coefficient and linearized Seebeck coefficient. Error bars show variations in the linearized calculation of both coefficients.

The linearized changes in voltage as a function of temperature difference are shown in Figure 4A. Each RTIL is associated with a distinct $\alpha_{lin}$, the highest being that of 4C-Cl${}^{-}$ or the 4 carbon ether linkage. The lowest Seebeck coefficient is associated with the longest-chain RTIL 10C-Br${}^{-}$ (10 carbon aliphatic). When introducing an ether linkage to N-3 on the aliphatic substituent, $\alpha_{lin}$ increases by a factor of 2 compared to its aliphatic constituent 4C Cl${}^{-}$. As the aliphatic chain length at the 3-position increases, $\alpha_{lin}$ is reduced.

From our experimental results, the alteration of halogens does not significantly change the measured α, therefore we are able to control for the ionic interactions between the opposing ions and observe how the repulsive interactions affect $\alpha_{lin}$. Higher Seebeck coefficients at small temperature changes are measured. At smaller temperature differences, the nonlinear characteristics may be influenced by the electronic double layer (EDL) and strong Coulomb interactions of RTILs. It has recently been reported that EDL arrangements in butyl and hexyl imidazolium-based RTILs with dominant electrostatic interactions between the aliphatic chains create multiple layers of fluid that alter the capacitance, which can indeed alter the voltage significantly at nanometer distances between electrodes [29,30,31]. With EDLs, the force required to reach a stable conformation increases as the chain length increases. The butyl substituent forms a monolayer of alternating ions, while the hexyl substituent forms a bilayer with the hydrocarbon chains aggregating with each other. While we anticipate that issues related to the EDL are present with all surfaces, EDLs are not expected to be the dominant role of the nonlinear Seebeck measurements, since our cell length is long in comparison [See Section 2].

There is a strong negative correlation between Seebeck coefficient and heat expansion of the RTIL, as shown in Figure 4B. We find that as chain length at the 3-position increases, the measured Seebeck coefficient decreases. We expect that as the chain length increases, the Coulombic repulsive energy increases. The Coulombic repulsive energy results in an overall decrease in lattice enthalpy, and decreases the attractive force between the intermolecular species [32,33,34]. Increased RTIL lattice enthalpy, increased attractive forces, and decreased chain lengths are expected to lower the heat expansion of RTILs. The experimental data at large temperature differences suggests that chain lengths should be minimized in order to increase $\alpha_{lin}$.

The presence of the activating group (oxygen) in 4C Cl${}^{-}$ ether is assumed to increase the lattice enthalpy by reducing the Coulombic repulsive interactions due to its increased electronegativity and donating capacity. Solid-state crystal structure analysis is very useful in determining the dependence on intermolecular forces and predicting volumetric heat expansion. Yet, studies have shown that the inherent intermolecular organization of imidazolium-based ILs alter the crystal state by forming either triclinic (for RTILs containing oxygen and/or high attractive forces) or monoclinic (weak attractive interactions) [35]. Furthermore, identifying key features of crystal structures can be very useful in predicting heat expansion trends. These species have also been reported in displaying a wider gap between the liquid and solid phase, due to the presence of an oxygen [36]. In other words, increased intra/inter-molecular interactions have a weak mesophasic characteristic, residing predominantly in the liquid phase. This apparent distinction between solid and liquid phase states also is dependant on chain length and solid/liquid crystal structures. The 10C-Br${}^{-}$ RTIL exists in an increased mesophasic state, rendering it a highly viscous material [17], hence a low measured $\alpha_{lin}$. In other words, extensive hydrophobic interactions along with its pi-pi stacking of imidazolium cations reduce its performance as a TEG fluid [37,38]. Increased RTIL lattice enthalpy, increased attractive forces, and decreased chain lengths are expected to lower the heat expansion of RTILs, which correlates with higher Seebeck responses.

## 4. Discussion and Conclusion

Our experimental results show a clear trend with a series of ionic liquids: when an RTIL expands, it does work, and the Seebeck coefficient drops. The degree to which the voltage drops is surprising: a few-percent change in volume leads to a drop in the Seebeck coefficient by at least a factor of 2. Since others have achieved Seebeck effects as high as −15 mV/K or higher [4], it is important to reconsider the thermodynamics of intra-molecular interactions.

As reported in the previous section, steric forces are expected to carry a significant role and to interfere with the ionic attraction simultaneously occurring between the charges, which reduce the measured voltage. This may explain why we do not measure that larger Seebeck coefficients correspond with greater asymmetry or longer-chain RTILs. In fact, we almost measure the opposite: The 10C Br${}^{-}$ (1-Decyl-3-methyllimidazolium Bromide) exhibits half the Seebeck coefficient than the 4C Br${}^{-}$ (1-Butyl-3-methylimidazolium Bromide). Our results lead us to ask two subsequent questions related to the thermodynamics of the TEG system: (1) Will the Seebeck coefficient be improved with longer-chain RTILs if the volume is fixed and (2) what is the thermodynamic role of steric forces with regards to the Seebeck voltage?

**Figure 5:**
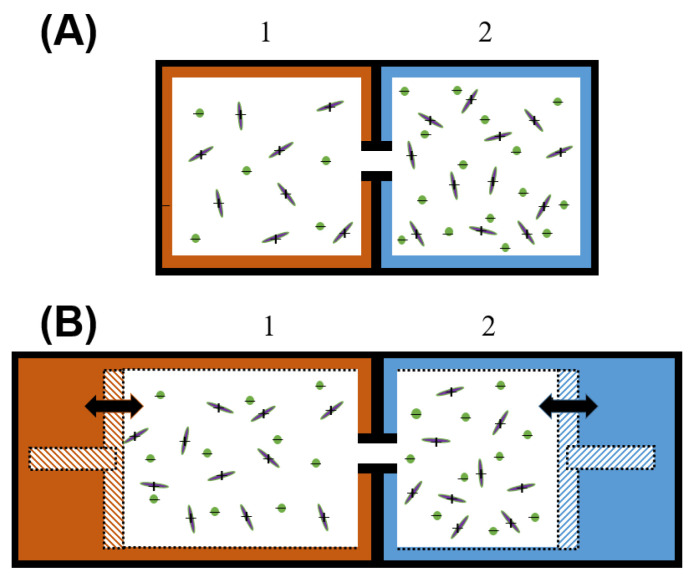
(**A**) Thermodynamic (N,V,T) isochoric model, where the volume of hot and cold cells (red and blue) are fixed; (**B**) Thermodynamic (N,P,T) isobaric model, where the cells have similar pressure but the volume of each cell is permitted to change.

Consider the two-chamber models for the thermodiffusion as employed previously [39] (Figure 5A,B). In Figure 5A,B the complementary positive and negative ions are drawn by rods and spheres, which represent different constituents with different internal energies associated with shape, mass, charge distributions, vibrational and rotational energies, among others. With two cells at two different temperatures, rods and spheres migrate between cells until equilibrium is achieved.

The Helmholtz energy (N,V,T) model may explain aspects of thermophoresis and thermodiffusion. This fixed-volume model predicts that constituents with higher mass are pushed towards the hot electrode; as smaller, lighter constituents with fewer degrees of freedom, the halides are more mobile. When there are electrical and chemical changes to the constituents, there is a shift in internal energy that must be further considered [40]. The net migration dynamics of rods and spheres are also affected by heat expansion, which can be accounted for in a Gibbs energy (N,P,T) model, where both chambers are allowed to expand, is shown in Figure 5B. We expect that as the RTIL expands, the electrostatic forces between complementary ions is reduced, which increases the mean free path or mobility of the RTIL aliphatic chain towards that for the anion, which leads to a reduction in the measured Seebeck voltage. Both intra- and inter-molecular interactions carry entropy conserving and entropy nonconserving roles in the expansion of a liquid [41], which are critical to understanding TEG fluid chemistry architectures and the fundamental thermodynamics and limits of TEG applications.

In conclusion, we report on the Seebeck coefficient for a range of RTILs and observe the role of heat expansion, which has not been systematically studied with TEGs before. The thermoelectric behavior of a series of viscous RTILs in a long, convection-minimal geometry is studied. Seebeck coefficients of −3 mV/K are measured with temperature differences of 10 K; however, these values drop rapidly when the temperature difference increases to 50 K. When the voltages as a function of temperature difference are linearized, we observe a strong negative correlation between heat expansion and Seebeck coefficient. When an RTIL expands, it does work, and the voltage produced in the temperature gradient decreases.

We show a negative correlation between thermal expansion and nonlinear Seebeck effect irrespective of chain length. Intra- and inter-molecular interactions of the RTIL are considered to carry a critical role, leading to heat expansion and reduced Seebeck coefficients. Shorter-chain RTILs exhibit lower heat expansion and are expected to exhibit higher thermoelectric responses. Since heat expansion reduces the measured Seebeck effect, we speculate that high-pressure, isochoric designs that prevent heat expansion will lead to higher measured Seebeck effects. Alternately, the RTIL design of ligands may also reduce steric intramolecular repulsion and improve measured Seebeck effects.
